# P-673. Respiratory Syncytial Virus (RSV) Hospitalizations During Off-Season Months Among Infants in US

**DOI:** 10.1093/ofid/ofae631.869

**Published:** 2025-01-29

**Authors:** Amy W Law, Jay Lin, Jennifer Judy, Alejandro D Cane, Sarah J Pugh

**Affiliations:** Pfizer, Inc., New York, New York; Novosys Health, GREEN BROOK, New Jersey; Pfizer, New York, New York; Pfizer, New York, New York; Pfizer, Inc., New York, New York

## Abstract

**Background:**

The CDC currently recommends two prevention options to help protect infants against RSV-associated lower respiratory tract disease during a typical RSV season, defined as October through March. However, annual and geographic variability in RSV seasonality exists. The study evaluated the burden of RSV hospitalizations occurring outside a typical season (i.e., April – September).

**Figure:**
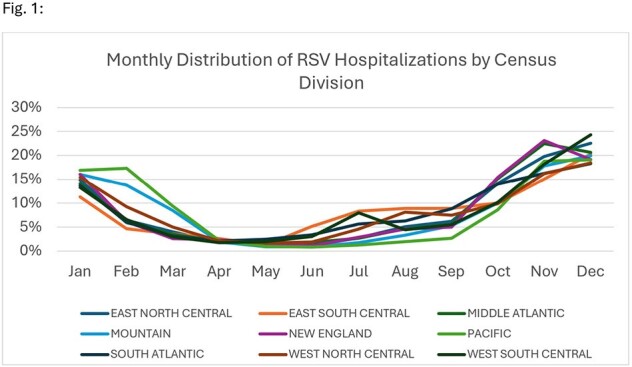

**Methods:**

Infants aged < 12 months old hospitalized with RSV were identified from the PINC AI Healthcare Database (7/1/2018 to 6/30/2023) for this descriptive cohort study. Because age is reported in years, a prior birth hospitalization record was required to determine age in months for RSV hospitalization. A surveillance year was defined by the period of July 1 of each year to June 30 of the following year. Monthly RSV hospitalizations were tallied for each of the 9 U.S. census divisions and by age group (< 3, 3-5, 6-8, 9-11 months). Seasonality of RSV cases among all infants < 1 year, regardless of the ability to identify age in months, was evaluated as a sensitivity analysis.

**Figure:**
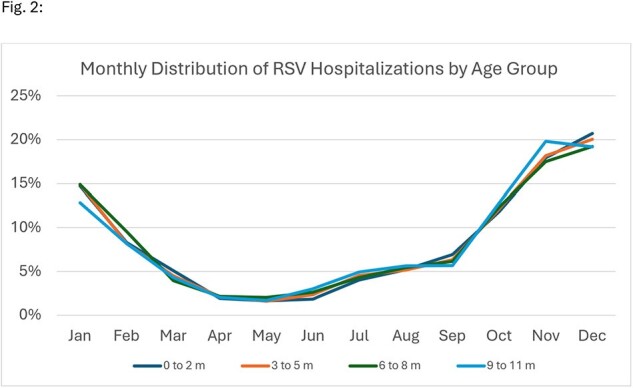

**Results:**

A total of 56,425 RSV hospitalizations were identified among infants < 1 year of age, of which, 36% (n=20,531) of patients had a birth record. RSV hospitalization counts during the 5 surveillance years were 4,574 (2018/19), 4,926 (2019/20), 452 (2020/21), 4,344 (2021/22), and 6,235 (2022/23). Overall, 22% (n=4,510) of RSV hospitalizations occurred during off-season months, with 83% of these off-season cases occurring in June – September across various divisions (Fig. 1). Of all RSV hospitalizations that occurred during off-season, 45.5% (n=2,054) were among infants < 3 months. Seasonal patterns were similar across age groups (Fig. 2). A sensitivity analysis showed consistent seasonal patterns among all infants < 1 year of age.

**Conclusion:**

This large-scale, real-world database analysis showed over 1 in 5 infants hospitalized with RSV were infected during off-season with varying rates by geography. The current recommendations may limit protection options for infants exposed to RSV outside the typical season.

**Disclosures:**

**Amy W. Law, PharmD**, Pfizer Inc.: Employee|Pfizer Inc.: Stocks/Bonds (Public Company) **Jay Lin, PHD, MBA**, Pfizer: Advisor/Consultant **Jennifer Judy, MS, PhD**, Pfizer: Employee|Pfizer: Stocks/Bonds (Public Company) **Alejandro D. Cane, MD, PhD**, Pfizer: Employee|Pfizer: Stocks/Bonds (Public Company)|Pfizer: Stocks/Bonds (Public Company) **Sarah J. Pugh, PhD, MPH**, Pfizer Inc.: Employee|Pfizer Inc.: Stocks/Bonds (Public Company)

